# Advances in Membrane-Bound Catechol-*O*-Methyltransferase Stability Achieved Using a New Ionic Liquid-Based Storage Formulation

**DOI:** 10.3390/ijms23137264

**Published:** 2022-06-30

**Authors:** Ana M. Gonçalves, Ângela Sousa, Augusto Q. Pedro, Maria J. Romão, João A. Queiroz, Eugénia Gallardo, Luís A. Passarinha

**Affiliations:** 1 CICS-UBI—Health Sciences Research Centre, University of Beira Interior, 6201-506 Covilhã, Portugal; ggmargarida@gmail.com (A.M.G.); angela@fcsaude.ubi.pt (Â.S.); jqueiroz@ubi.pt (J.A.Q.); 2 Associate Laboratory i4HB-Institute for Health and Bioeconomy, Faculdade de Ciências e Tecnologia, Universidade NOVA, 2819-516 Caparica, Portugal; mjr@fct.unl.pt; 3 UCIBIO-Applied Molecular Biosciences Unit, Departamento de Química, Faculdade de Ciências e Tecnologia, Universidade NOVA de Lisboa, 2829-516 Caparica, Portugal; 4 CICECO-Aveiro Institute of Materials, Chemistry Department, University of Aveiro, Campus Universitário de Santiago, 3810-193 Aveiro, Portugal; apedro@ua.pt; 5 Laboratório de Fármaco-Toxicologia, UBI Medical, Universidade da Beira Interior, 6201-506 Covilhã, Portugal

**Keywords:** membrane-bound catechol-*O*-methyltransferase, Design of Experiments, enzymatic activity, stability, ionic liquids

## Abstract

Membrane-bound catechol-*O*-methyltransferase (MBCOMT), present in the brain and involved in the main pathway of the catechol neurotransmitter deactivation, is linked to several types of human dementia, which are relevant pharmacological targets for new potent and nontoxic inhibitors that have been developed, particularly for Parkinson’s disease treatment. However, the inexistence of an MBCOMT 3D-structure presents a blockage in new drugs’ design and clinical studies due to its instability. The enzyme has a clear tendency to lose its biological activity in a short period of time. To avoid the enzyme sequestering into a non-native state during the downstream processing, a multi-component buffer plays a major role, with the addition of additives such as cysteine, glycerol, and trehalose showing promising results towards minimizing hMBCOMT damage and enhancing its stability. In addition, ionic liquids, due to their virtually unlimited choices for cation/anion paring, are potential protein stabilizers for the process and storage buffers. Screening experiments were designed to evaluate the effect of distinct cation/anion ILs interaction in hMBCOMT enzymatic activity. The ionic liquids: choline glutamate [Ch][Glu], choline dihydrogen phosphate ([Ch][DHP]), choline chloride ([Ch]Cl), 1- dodecyl-3-methylimidazolium chloride ([C12mim]Cl), and 1-butyl-3-methylimidazolium chloride ([C4mim]Cl) were supplemented to hMBCOMT lysates in a concentration from 5 to 500 mM. A major potential stabilizing effect was obtained using [Ch][DHP] (10 and 50 mM). From the DoE 146% of hMBCOMT activity recovery was obtained with [Ch][DHP] optimal conditions (7.5 mM) at −80 °C during 32.4 h. These results are of crucial importance for further drug development once the enzyme can be stabilized for longer periods of time.

## 1. Introduction

Proteins are sensitive biomolecules that often require great care during manipulation to ensure that they remain intact and fully active [1]. This is especially true for enzymes, who present assayable biological activities and whose maintenance is generally of prime importance, both during protein purification and for subsequent studies of function and structural analysis [1]. The characterization of individual proteins often requires that they are produced in quantities that go far beyond their abundance in the cell, as they are recombinantly produced and isolated in an environment that is devoid of potentially stabilizing factors [2]. It is not surprising that many protein samples show a loss of function and a reduced stability in standard sample buffer conditions [2]. Therefore, it is crucial to identify the additives that are essential to recovering the integrity and ensuring the activity of the enzyme [2]. 

During the last decades, catechol-*O*-methyltransferase (COMT) has been implicated in several human diseases, including different neurodegenerative disorders such as schizophrenia and Parkinson’s Disease (PD) [3,4], estrogen-induced cancers [5,6,7], or cardiovascular diseases [8]. However, the best-documented implication is the important role that COMT plays in PD, whose most effective treatment remains dopamine replacement therapy with levodopa, together with an inhibitor of the aromatic amino acid decarboxylase and a COMT inhibitor [4]. In mammals, COMT is present in two isoforms, a membrane-bound isoform (MBCOMT) associated with the rough-endoplasmic reticulum and a soluble form (SCOMT) present in cytoplasm [4]. However, an increasing relevance has been assigned to MBCOMT due to its high abundance in the human brain and its higher affinity for catechol substrates when compared to soluble isoforms [4,9].

Thus, the development of combined upstream and downstream strategies for obtaining high quantities of biologically active, stable, and pure enzyme fractions remains an essential goal, with their application envisaged as a therapeutic protein or to be used to conduct structural studies. 

A major bottleneck was raised from preliminary chromatographic studies that intended to obtain COMT in a purified form, which identified this enzyme as highly unstable and described it as extremely labile, as it rapidly loses its biological activity during recovery and storage [10,11]. 

Usually, an increase in protein stability is obtained by the addition of cosolvents using a mechanism that is related to the shift of protein conformations toward more compact and ordered states as well as the inhibition of partial unfolding that leads to protein aggregation [12]. So, over the years, COMT has been stabilized through the application of specific compounds capable of increasing its thermodynamical stability which prevents its denaturation and activity losses. The stabilizer formulations that were applied included compounds that are reducing agents such as dithiothreitol (DTT), cysteine, sugars such as sucrose and trehalose, and cryoprotectors such as glycerol [11,13,14,15]. Protein stability and solubility depends on its interface with the solvent. Ions that strongly interact with water are called “kosmotropic” (structure makers) and if the interaction is weak, the ions act in a “chaotropic” (structure breaking) manner [16,17]. Small ions (kosmotropes), due to their high charge densities, break hydrogen bonds, and large ions (chaotropes) have low charge densities and are largely hydrogen bonded [16,18]. Indeed, the kosmotropic agents are known to stabilize intra-molecular bonds, [19] while reducing agents, polyols, and sugars usually affect protein stability and have been shown to affect SCOMT stability [15]. Osmolytes, such as trehalose, protect proteins from denaturation *in vivo* and have also been described as promoters of protein folding and refolding [19]. The general mechanism for stabilization via the use of osmolytes is believed to be achieved by changing the protein hydration, via the exclusion from the hydration layer of the protein, resulting in a shift in the protein’s melting temperature and thus increasing the energy needed to denature proteins [19]. On the other hand, conserved cysteines usually play a role in the formation of intramolecular and intermolecular disulfide bonds [20,21] that are essential for maintaining the native three-dimensional structure. Polyols, such as glycerol, have also been applied for thermostabilizing enzymes [22] by mechanisms related to the excluded the solvophobic effect, which leads to protein conformations with a minimal solvent-accessible surface area [12].

More recently, ionic liquids (ILs) have also shown their unique capability to preserve protein stability, folding, and to prevent protein aggregation due to the diverse ion combinations that provide them with different physicochemical properties [23,24,25]. This vast range of cation/anion combinations affords a different balancing of intermolecular interactions [24]. The selection of ion combinations and hydrophobic and polar interactions from each amino acid leads to enzyme stability through the application of an IL [24]. ILs could be classified in two broad categories, the atropic ILS (AILs) and the protic ILS (PILs). The difference is a proton transference between a Bronsted acid to a Bronsted base that can be used to build a hydrogen-bonded network in PILS. The cations used are predominantly nitrogen-based (ammonium ions including primary, secondary, and tertiary) coupled to anions such as carboxylate, alkylsulfonate, and nitrate. On the other hand, AILs include a broad set of cations such as imidazolium, 1-alkylpyridinium, and quaternary ammonium ions. Usually, these cations are coupled with chloride, tetrafluoroborate, or carboxylate [25].

Choline based ILs have been widely used in catalysis and biomedical, diagnostic, and tissue engineering fields due to their enhanced biodegradability and biocompatibility properties [26]. Moreover, it was reported that choline dihydrogen phosphate, an example of a combination between a chaotropic cation with a kosmotropic anion, were shown to stabilize cytochrome c [27,28] and lysozyme [29] during long storage periods. 

On the other hand, imidazolium based ILs with different cationic alkyl chain lengths have been employed as co-solvents or additives to suppress protein aggregation and to promote protein refolding and crystallization. [30,31,32]. For instance, [C4mim]Cl was reported as being capable of dissolving cellulose, once the chloride wins the hydrogen bonding competition for cellulose’s OH-groups against the intramolecular hydrogen bond network [33]. Moreover, Lange et al. studied the effect of Imidazolium based ILs on lysozyme and the single-chain antibody fragment ScFvOx and verified that *N*′alkyl and *N*′(hydroxyalkyl)-*N*-methylimidazolium chlorides with alkyl chain lengths of two to six carbon atoms acted as refolding enhancers, and in some cases, were better than L-arginine hydrochloride, the most widely used additive for protein renaturation [34]. Regarding anion paring, it was shown by Buchfirk et al. that the variation in the anion had a tremendous effect on the renaturation of the recombinant activator (rPA), with anions such as chloride leading to a reduction in the refolding yields [35]. 

Therefore, it is desirable to systematize the ion effects on enzyme properties such as activity, stability, and the retaining of the structure during refolding processes. To the best of our knowledge, there are no studies reporting the application of ILs for the stabilization of COMT. Therefore, to investigate the effect of these compounds upon the stability and activity of hMBCOMT, the enzymatic biological activity was measured in the presence of these ILs: choline glutamate [Ch][Glu], choline dihydrogen phosphate ([Ch][DHP]), choline chloride ([Ch]Cl), 1- dodecyl-3-methylimidazolium chloride ([C12mim]Cl), and 1-butyl-3-methylimidazolium chloride ([C4mim]Cl). Herein, we have developed a DoE to optimize the most suitable conditions for the storage of recombinant human MBCOMT (hMBCOMT) obtained from *Komagataella pastoris* (*K. pastoris*) lysates, measured as the ratio between the hMBCOMT biological activity levels before and after the storage of the samples under each experimental condition. Overall, it was observed that the additives cysteine, glycerol, trehalose, and the ionic liquid [Ch][DHP] influenced the stability of the enzyme and that its optimal concentrations should be determined to ensure the maintenance of hMBCOMT’s biological activity during storage. The storage temperature was considered −80 and 4 °C, and storage periods were 24, 48, and 72 h. These parameters and the [Ch][DHP] concentration were selected to achieve the maximum recovery of hMBCOMT biological activity after storage.

## 2. Results and Discussion

A high protein concentration and long-term stability are essential requirements in a wide range of applications, from the preparation of formulations in pharmaceuticals to general biochemical studies, and particularly in the field of structural biology [36]. In addition, the efficient large-scale production of recombinant proteins depends on the careful conditioning of the protein during its production, isolation, and purification [2]. A low protein stability leads to decreased purification yields as a result of protein degradation, precipitation, and folding instability [2]. The preparation of a concentrated and stable protein sample is often a difficult task once the proteins start to frequently aggregate or precipitate at higher concentrations, and/or are subject to spontaneous proteolytic degradation [36]. It is often necessary to go through several iterations of trial-and-error to optimize the homogeneity, stability, and solubility of the target protein [2].

The stability of COMT enzymatic preparations, as well as other proteins [37], is affected by physical-chemical parameters such as pH and temperature. According to some authors, COMT is an extremely unstable enzyme and loses 50–70% of its activity in less than 24 h even at 4 °C [38]. However, proteins are thermodynamically stable within a certain temperature range. In this work, different stabilizers were added to hMBCOMT lysates to increase its thermal stability and help maintain its native conformation after storage at a specific temperature and time. 

### 2.1. Preliminary Studies for hMBCOMT Stabilization

As a protein is recombinantly produced, several concerns regarding its recovery and isolation arise, since it can be influenced by pH, buffers, and additives such as salts, sugars, amino acids, and more recently, the presence of ionic liquids. Therefore, an examination of the influence of these compounds in the protein activity is crucial. 

From previous work performed by our research group, it was established that the ideal buffer composition to perform the recovery of hMBCOMT from *K. pastoris X-33* cells was constituted by 150 mM NaCl, 10 mM DTT, 1 mM MgCl_2_, and 50 mM Tris at pH 8.0 [39]. The salt concentration, the pH (between 3 and 8), and the introduction of the reducing agent, to prevent the oxidation of cysteine residues and consequently avoid protein aggregation, intends to mimic the physiological conditions and ensure protein activity [40]. Moreover, the addition of MgCl_2_ provides the magnesium ion, one of the cofactors required for enzyme activity. Therefore, unless otherwise stated, the stabilizers in the study were solubilized for all the experiments in this base buffer, with the exception of DTT, which at the concentration used overlaps the peak of metanephrine, the product that quantifies the activity of the enzyme obtained by HPLC with coulometric detection [41].

Taking into consideration the different additives used to stabilize COMT (cysteine, sucrose, trehalose, and glycerol) and their broad range of concentrations (between 5 to 250 mM) [13,14,15], stabilization studies proceeded with the additives cysteine, trehalose, and glycerol at compatible concentrations that do not compromise biointeractions and structural studies. Sucrose and DTT were not considered for further studies since both molecules interfere with metanephrine quantification. 

**Figure 1:**
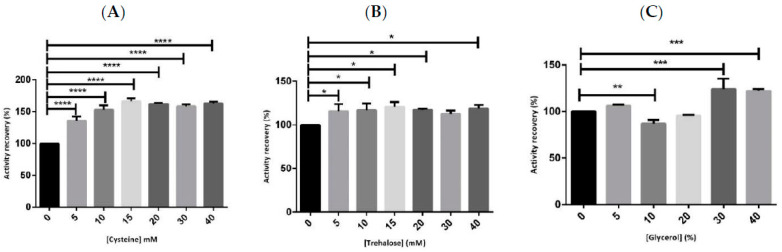
Graphical representation of hMBCOMT activity recovery without the addition of additives in comparison to hMBCOMT in contact with the additives: (**A**) Cysteine; (**B**) Trehalose; (**C**) Glycerol after 12 h at 4 °C. (*p*-value * <0.05; ** <0.01; *** <0.001; **** <0.0001).

The first stabilizer to be evaluated was cysteine in a concentration range between 5 to 40 mM, as shown in Figure 1A. In particular, it was found that with the application of low concentration values of 15 mM, the protein activity could be maintained and even increased in comparison to our blank (hMBCOMT lysate without the addition of stabilizers) after a period of 12 h at 4 °C. Taking into consideration the previous concentrations used, a range from 140 mM to 250 mM, a 10-fold reduction in cysteine concentration could be achieved without compromising the activity of the protein [11,15].

Concerning trehalose, the studied concentrations were once again maintained between 5 and 40 mM, as shown in Figure 1B. For this additive, it was demonstrated that all the concentrations used were capable of maintaining the activity of hMBCOMT after 12 h at 4 °C with minor changes among them. 

In terms of glycerol, the percentage range of the study went from 5 to 40%, as shown in Figure 1C. Based on the results, the amounts of 5, 30, and 40% seem to be ideal for maintaining the biological activity of hMBCOMT, with the higher values slightly increasing the percentage of the activity recovery. However, for high values of glycerol, interferences were detected in the obtained chromatograms for the determination of the protein activity (data not shown). Concerning the *p*-values, the results were highly significant for cysteine (*p*-value < 0.0001) and glycerol (*p*-value < 0.01 and *p*-value < 0.001). In summary, comparing the three additives, they were all capable of maintaining and even increasing the values of hMBCOMT activity recovery, with cysteine presenting a major impact on the input. This fact is probably due to cysteine’s capacity to form reversible covalent cross-links in proteins that influence its stability and activity [21]. Once our goal was to reduce the stabilizer concentrations used without compromising hMBCOMT activity for further structural studies, we established the additive concentrations of cysteine, trehalose, and glycerol to be 15 mM, 5 mM, and 5%, respectively.

Recently, ionic liquids (ILs) have been considering as novel biocompatible co-solvents for proteins [42,43]. Their uniquely tailored chemical and physical properties set ILs in the center field of various protein-based applications such as crystallization, separation, extraction, solubilization, and stabilization [44,45]. Thus, our group decided to study two major classes of ILs. The first was the choline-based IL, which was extensively studied to reveal whether it promotes or inhibits protein aggregation and protein stabilization, with its outcome being IL concentration dependent [28,46,47]. The second was the imidazolium-based IL, which was employed as a co-solvent or an additive for protein crystallization as shown by the group in the stabilization of periplasmic molybdenum aldehyde oxidoreductase (PaoD) protein in the presence of [C4mim]Cl, with its effect being alkyl chain-length dependent [32,48]. Moreover, as shown in Table 1, beyond the study of the two different cation classes, we also analyzed a wide range of IL concentrations, between 5 to 500 mM and with different anion paring, since both cationic and anionic parts of IL can play an important role in protein stabilization [35].

In the Table 1 analysis, regarding the imidazole based IL, it was observed that an increment in the IL alkyl chain’s length had a negative effect on the percentage of hMBCOMT biological activity recovery for all the study concentrations, with [C12mim]Cl being the only IL with which we were unable to assess hMBCOMT activity. It seems that hydrophobic imidazolium cations with longer alkyl chains—as is the case of [C12mim]Cl in comparison with [C4mim]Cl—increased the destabilization of the protein, probably due to the stronger surfactant, which has an ulterior effect on the protein activity [43,49]. 

Concerning the choline-base IL, all three ILs [Ch][Glu], [Ch][DHP], and [Ch]Cl are capable of maintaining and increasing the percentage of hMBCOMT activity recovery, with the optimal reported values being IL concentration dependent. The IL [Ch][Glu] presented the highest percentage of hMBCOMT activity recovery with the maximum tested concentration of 250 mM. On the other hand, for [Ch]Cl and [Ch][DHP], the best results were obtained when lower concentrations of 5 to 10 mM and 10 to 50 mM were used, respectively, with [Ch][DHP] presenting a 99% hMBCOMT activity recovery increase when compared with hMBCOMT without the IL. Taking into consideration the different anions study, it seems that DHP presents a stronger interaction with hMBCOMT, capable of stabilizing the protein with lower concentration values during 12 h at 4 °C. 

**Figure 2:**
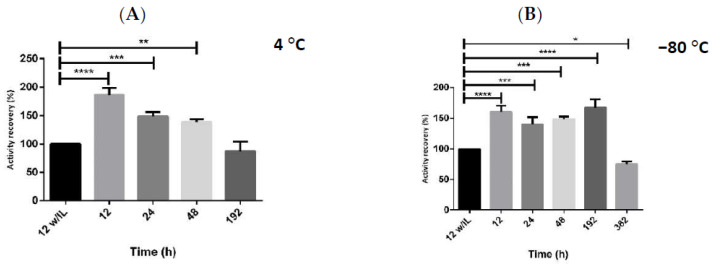
Graphical representation of the activity recovery of the protein hMBCOMT lysate in comparison to hMBCOMT in contact with 10 mM of [Ch][DHP] when storage at (**A**) 4 and (**B**) −80 °C. (*p*-value * <0.05; ** <0.01; *** <0.001; **** <0.0001).

In addition, if we compare the best results obtained for each cation-based class, [Ch][DHP] and [C4mim]Cl, the first one presented higher increments in the biological activity recovery (199%), achieving almost double of the initial activity obtained for the hMBCOMT lysates, even when the protein was stored for longer periods, as shown in Figure 2. This behavior was also observed for proteins such as cytochrome c [28] and lysozyme [29]. Thus, through the use of [Ch][DHP], we were able to pass from a half percentage of hMBCOMT activity recovery in the first 24 h to be able to prolong its enzymatic activity during 192 h, at −80 °C storage, with the highest recovery activity of 150%.

It was reported by Zaks and Klibanov that the solvent polarity is related to the protein solubility and water association between the solvent and protein. Moreover, it was demonstrated that the hydrophobicity can be quantified by polarity while β describes the solvents ability to donate electron density to form a hydrogen bond with the protons of a solute, which is an important parameter to take into consideration for stabilizing enzymes [50]. ILs that can support enzyme activity have anions with lower β values, since the interference of the IL anions with the internal hydrogen bonds of the protein are minimized [51]. Such a finding is in concordance with our results, which show that the ionic liquid [Ch][DHP], with a low β value, presents the best activity recovery output [52]. 

Moreover, regarding the ILs biocompatibility, it was demonstrated that imidazolium-based ILs are less biocompatible than cholinium-based ILs, since [Ch][DHP] is considered “practically harmless” and has already been employed in the development of stable therapeutic antibody formulations [26,47].

### 2.2. Enzymatic Stability with Multicomponent Buffer

**Figure 3:**
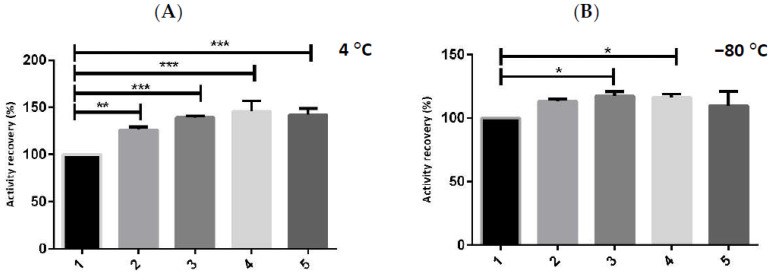
A graphical representation for both (**A**,**B**) of the activity recovery of the protein hMBCOMT lysate: 1—base buffer; 2—base buffer with 10 mM of [Ch][DHP]; 3—buffer 2 with 15 mM of cysteine; 4—buffer 3 with 5 mM of trehalose and 5—buffer 4 with 5% of glycerol, hMBCOMT lysate storedat 4, and −80 °C for 12 h. (*p*-value * <0.05; ** <0.01; *** <0.001).

To study the synergistic effect of each additive on hMBCOMT activity, each molecule was added separately, and a multicomponent buffer formulation was performed through the gradual addition of each additive to the buffer base, which consisted of: 150 mM NaCl, 1 mM MgCl_2,_ and 50 mM Tris at pH 8.0, as shown in Figure 3. 

The results demonstrate that the interaction of all the additives ([Ch][DHP], cysteine, trehalose, and glycerol) in the base buffer formulation with hMBCOMT maintains and even enhances the protein stability at both storage temperatures (4 and −80 °C) for a period of 12 h. In particular, the final multicomponent buffer composed of 150 mM NaCl, 1 mM MgCl_2_, 50 mM Tris at pH 8, 10 mM of [Ch][DHP], 15 mM of cysteine, 5 mM of trehalose, and 5% of glycerol, increases the enzymatic activity most notably when the lysate sample is stored at 4 °C, *p*-value < 0.001 (Figure 3A, sample 5), which is crucially important for performing future structural and functional studies.

### 2.3. Storage Stability of hMBCOMT with DoE

Considering that our goal was to improve the stability of hMBCOMT upon storage, three input parameters were defined, namely, the concentration of the ionic liquid used in the buffer formulation, and the time and temperature of storage. From previous studies (data not shown), the parameters were explored with a defined range of 7.5 to 12.5 mM for the concentration of [Ch][DHP], 24 to 72 h was the time of storage, and 4 to −80 °C was the storage temperature. The selected output was the percentage of hMBCOMT activity recovery, and the CCF design was chosen for this work. The model proposed 22 experiments to be performed with different input conditions, with 3 replicates of the central point, as indicated in Table 2. Each individual experiment was conducted according to the storage conditions proposed by the DoE. The percentage of hMBCOMT bioactivity recovery was determined by a calculation of the ratio between the specific activity of hMBCOMT without stabilizers upon 24, 48, and 72 h at 4 and −80 °C (our blanks), and the specific activity of hMBCOMT at the defined conditions. As it can be seen in Table 2, run 1, to obtain a higher value of hMBCOMT activity recovery, a low concentration of [Ch][DHP] (7.5 mM) should be used and stored at −80 °C for 24 h to obtain an activity recovery of 148.2%.

### 2.4. Model Generation and Statistical Analysis

After the accomplishment of all the experiments proposed by the CCF design and an assessment of the outputs, a statistical analysis was performed using design-Expert software. In Table 3, the statistical coefficients obtained for the percentage of hMBCOMT activity recovery are presented, which were applied to determine whether the statistical model generated from these experiments is valid and fits the data. Thus, by analyzing the coefficient of determination (R^2^), which provides information regarding the fitness of the output statistical model to the data and the adjusted R^2^, representing the theoretical values being adjusted to the experimental data, the obtained values of 0.9770 (close to 1) and 0.9629 suggest that the model fits the data and the output presents a valid adjustment of R^2^ since it only decreases by 0.0141 [53,54] compared to its R^2^. Regarding the information concerning the suitability of the model for predicting new data, the predicted R^2^ with a value of 0.9074 highlights the predictive power of the present model. Finally, taking into consideration the measurement of the signal-to-noise ratio, provided by the adequate precision, and presenting a value of 27.056 (greater than four), it is suggested that the model can be used to navigate the design space [55]. 

Observing all these coefficients, the quadratic model was chosen to proceed with the statistical analysis of this output. To further prove the validity of the DoE, ANOVA analysis was performed. Table 4 shows the model significance for the output, including all the parameters used in this model coupled with the corresponding lack of fit. A valid model must present a significant value for the model (*p*-value < 0.05) and a non-significant value for the lack of fit (*p*-value > 0.05), thus suggesting the model data are significant and fit [55]. According to Table 4, the model is significant and does not present a significant lack of fit. Moreover, factors A, B, and C, as well as the interaction of the factors B and C, are significant. On the other hand, the interactions between factors A and B, A and C, as well as A^2^ and B^2^ do not present significance. In summary, a good and valid statistical model was achieved for this output.

### 2.5. Model Generation and Statistical Analysis

To evaluate the main effects that each input presents towards the percentage of hMBCOMT activity recovery, a coded multiple regression equation was generated by Design-Expert software. In this equation, the signal behind each factor indicates a positive or negative effect in the response. Equation (1) presents the output regression equation, where A is the concentration of the ionic liquid [Ch][DHP] (mM), B is time (h), and C is the temperature of the storage (°C).

(1) $$\begin{array}{ll} \%\;\mathrm{hMCOMT}\;\mathrm{activity}\;\mathrm{recovery} & =104.91-7.82A-17.42B-24.19C+2.20\mathrm{AB}+1.53\;\mathrm{AC}\\ & -\;7.42\;\mathrm{BC}\;+\;0.12A^{2}\;-\;3.82B^{2} \end{array}$$

Through the analysis of Equation (1), it can be deduced that the concentration of [Ch][DHP], the time, and the temperature of storage negatively affect enzyme activity and consequently the stability. Therefore, higher values of A, B, and C lead to lower values of the percentage of hMBCOMT activity recovery. Moreover, if the concentration of [Ch][DHP] is analyzed in relation to the time or the temperature of storage, a positive effect will be observed in the output response. However, if we analyzed the interaction of the time with the storage temperature, a negative effect is expected to be observed in the output response. The factor C^2^ is not present in the Equation (1), since it was defined as categorical to allow for the use of feasible temperature values in the laboratory.

### 2.6. Output Optimization and Model Validation

After the validation of the statistical model and the determination of the effect that each factor has on the output, the conditions of each input to reach the optimal point (maximizing activity recovery) were predicted. The Design-Expert software suggested that the combination of a low concentration of [Ch][DHP] 7.5 mM incorporated in the multicompetent buffer defined (150 mM NaCl, 1 mM MgCl_2_, and 50 mM Tris pH 8; 15 mM of cysteine, 5 mM of trehalose, and 5% of glycerol) for a period of 32.4 h at −80 °C are considered the ideal conditions to obtain a maximized percentage of hMBCOMT activity recovery between 136.787 and 152.961% of the confidence interval for the validation of the optimal point. These conditions were applied in three independent experiments and the average of the resulting outputs provided a recovery of 138.9 ± 2.5%. This value is within the confidence interval provided by the Design-Expert software, where the output is considered valid, according to Table 5.

## 3. Materials and Methods

### 3.1. Instruments, Softwares, Materials and Reagents

HPLC model Agilent 1260 system equipped with an autosampler and quaternary pump coupled to an Hypersil^TM^ BDS C18 analytical column (250 × 4.6 mm i.d. 5 µm) from Thermo Fisher Scientific (Waltham, MA, USA) and an ESA Coulochem III (Milford, MA, USA) coulometric detector was obtained from Agilent (Santa Clara, CA, USA).

Prism 6 was obtained from GraphPad Software Inc (San Diego, CA, USA) and Design-Expert version 11 was obtained from Stat-Ease (Minneapolis, MN, USA).

Ultrapure reagent-grade water for HPLC was obtained with a Milli-Q system from Merck KGaA (Darmstadt, Germany). Also, glass beads (500 µm), glucose, S-adenosyl-_L_-methionine (SAM), epinephrine (bitartrate salt), DL-methanephrine hydrochloride, citric acid monohydrate, sodium octil sulfate (OSA), cysteine (L-), trehalose, biotin, protease inhibitor cocktail and 1-butyl-3-methylimidazolium chloride ([C4mim]Cl), were purchased from Merck KGaA (Darmstadt, Germany). Zeocin and Pierce BCA Protein Assay Kit was obtained from Thermo Fisher Scientific (Waltham, MA, USA). Agar was obtained from Pronadisa (Basel, Switzerland). Yeast nitrogen base and yeast extract were obtained from Himedia (Thane West, Maharashtra, India). Peptone was obtained from Becton, Dickinson, and Company (Franklin Lakes, NJ, USA). The 1-dodecyl-3-methylimidazolium chloride ([C12mim]Cl) and choline dihydrogen phosphate ([Ch][DHP]) were purchase from IoLiTec (Heilbronn, Deutschland). Choline glutamate [Ch][Glu] and choline chloride ([Ch]Cl), were provided by (Bio)molecular Structure and Interactions by NMR of UCIBIO, NOVA University, Lisbon. Dithiotreitol (DTT), dipotassium phosphate (K_2_HPO_4_), perchloric acid, and sodium acetate anhydrous (NaH_2_PO_4_) were obtained from Panreac (Barcelona, Spain). Monopotassium phosphate (KH_2_PO_4_) and magnesium chloride (MgCl_2_) were obtained from Chem-Lab (Zedelgem, Belgium). Methanol, glycerol, and acetonitrile were obtained from VWR (Radnor, PA, USA). All chemicals used were of analytical grade, commercially available, and used without further purification.

### 3.2. Recombinant hMBCOMT Biosynthesis and Recuperation

The production of the human recombinant MBCOMT was performed according to the procedure described by Pedro et al. [39]. Briefly, transformed *K. pastoris* X33 with the expression vector was grown for 72 h at 30 °C in yeast extract peptone dextrose (YPD) medium plates with 200 µg/mL of Zeocin. A single colony was inoculated in 100 mL of buffered minimal glycerol medium (BMGH) in 500 mL shake flasks. Cells were grown at 30 °C and 250 rpm to a cell density of 600 nm (OD600) of 6.0 units. Afterwards, an aliquot was introduced into 100 mL of buffered minimal methanol medium (BMMH) in 500 mL shake flasks, with an initial OD600 fixed to 1.0 unit. After 24 h of growth at 30 °C and 250 rpm, cells were collected by centrifugation (1500× *g*, 10 min, 4 °C). The cells were resuspended in lysis buffer (150 mM NaCl, 10 mM DTT, 50 mM Tris pH 8, 1 mM MgCl_2_) supplemented with protease inhibitor cocktail. Then, a mechanical treatment with glass beads (7 cycles of vortexing for 1 min with 1 min of interval on ice) was applied to disrupt the cells. Cell suspensions were centrifuged (500× *g*, 5 min, 4 °C). The supernatant was removed, and the pellet obtained was resuspended in the same lysis buffer, without DTT. The samples were stored at 4 °C until use for further assays. 

### 3.3. hMBCOMT Stabilization Studies at Different Time and Temperatures

The chosen stabilizers were added separately and in a range of concentrations to the hMBCOMT protein lysates (normalized to a protein content of 1 mg mL^−1^), as shown in Table 6. Experiments were performed primarily at 4 °C with the specified stabilizer concentrations during 12 h. Enzymatic hMBCOMT activity levels were evaluated by the capacity of the enzyme to convert epinephrine to metanephrine as described previously [41]. The percentage of the enzyme activity recovery was determined by the ratio between the specific activity at 12 h without stabilizers and the specific activity at 12 h with the stabilizers at the reported concentration. Further, we conducted, through the same methodology, an assay to evaluate the concentrations of [Ch][DHP] at 4 °C and −80 °C during the maximum period of time, ensuring the activity. All data analysis was performed using Prism 6 (GraphPad Software Inc. San Diego, CA, USA).

### 3.4. Design of Experiments (DoE) 

DoE was used to optimize the hMBCOMT stability conditions upon storage through the application of a CCF model. For this purpose, the input concentration of the ionic liquid [Ch][DHP] (mM), the time (h), and the temperature of storage (°C) corresponds to A, B, and C, respectively. The percentage of hMBCOMT activity recovery was the output to be maximized. The inputs were studied at three levels (−1; 0; and +1). The software Design-Expert version 11 was applied to the CCF model and proposed a total of 22 experiments, considering three replicates of the central point. After performing all the experiments and assessing the respective outputs, the results were included in the DoE software and all statistical analyses were performed to validate the model suitability. The generalized second-order polynomial model equation used in the response surface analysis is presented below (Equation (2)):

(2) $$Y=\beta_{0}+\beta_{1}X_{1}+\beta_{2}X_{2}+\beta_{3}X_{3}+\beta_{11}+\beta_{22}X_{12}^{2}+\beta_{33}X_{3}^{2}+\beta_{12}X_{1}X_{2}+\beta_{13}X_{1}X_{3}+\beta_{23}X_{2}X_{3}$$

The optimal point to maximize the activity recovery of hMBCOMT was predicted by the DoE and validated by performing three assays as replicates of proposed conditions. Finally, the average in percentage activity recovery obtained in these replicates was within the confidence interval (CI) of 95% provided by the DoE for the chosen optimal point.

### 3.5. Output Determination

The percentage of the activity recovery of hMBCOMT was analyzed through the ratio between the enzymatic specific activity without stabilizers and the hMBCOMT specific activity with the stabilizers at the reported concentration, time, and temperature. All data analysis was performed using Prism 6 (GraphPad Software Inc., San Diego, CA, USA).

## 4. Conclusions

The present work focused on the maintenance of the hMBCOMT’s stability for situations when the protein needs to be stored for long periods of time. To achieve this goal, the manipulation of temperature, storage time, and the concentration of the chosen additives ([Ch][DHP], cysteine, trehalose, and glycerol) was performed. Moreover, a DoE where the IL concentration of [Ch][DHP], time, and the temperature of storage was also implemented and proved to be suitable for successfully discerning the effect of the chosen variables. 

The buffer defined in the present work led to the maintenance of hMBCOMT activity for up to 32.4 h, storage at −80 °C, and even to an increase of approximately 40% when related to its original biological activity. To the best of our knowledge, this is the first study performed using these optimal conditions, and in particular the use of ILs, that has led to the maintenance of hMBCOMT biologic activity for a long period of time. These results are of critical importance because, until now, this type of enzyme stability has never been accomplished. This achievement is a great step to obtaining new pharmaceutical drugs to mitigate Parkinson’s disease.

## Figures and Tables

**Table 1:** hMBCOMT activity recovery in contact with different ILs in comparison with enzyme lysate samples without the IL understudy.

% of hMBCOMT Activity Recovery					
[IL] (mM)	[Ch][Glu]	[Ch][DHP]	[Ch]Cl	[C12mim]Cl	[C4mim]Cl
5	127% ± 4.15	126% ± 1.83	131% ± 2.77	-	128% ± 1.38
10	132% ± 6.23	199% ± 10.97	132% ± 8.88	-	130% ± 10.96
50	135% ± 6.79	199% ± 2.96	91% ± 1.37	-	101% ± 31.72
125	114% ± 2.3	101.37% ± 0.75	105% ± 0.49	-	90% ± 5.79
250	142% ± 2.9	-	106% ± 0.35	-	83% ± 2.76
500	Not tested	-	110% ± 5.37	-	-

- hMBCOMT without enzymatic activity.

**Table 2:** Bioactivity recovery of hMBCOMT obtained for each condition of the DoE tested.

Assay Number	Ionic Liquid Concentration (mM)	Time (h)	Temperature (°C)	% of hMBCOMT Activity
1	7.5	24	−80	148.2% ± 1.9
2	7.5	24	4	106.66% ± 3.6
3	10	24	−80	142% ± 0.44
4	10	24	4	98.7% ± 0.8
5	12.5	24	−80	122.9% ± 0.7
6	12.5	24	4	93.09% ± 1.8
7	7.5	48	−80	135% ± 1.9
8	7.5	48	4	91.27% ± 1.5
9	10	48	−80	125.9% ± 5.3
10	10	48	−80	127.6% ± 3.1
11	10	48	−80	128.5% ± 3.4
12	10	48	4	88.14% ± 4.2
13	10	48	4	81.4% ± 2.9
14	10	48	4	79.2% ± 0.14
15	12.5	48	−80	114.7% ± 1.0
16	12.5	48	4	77.9% ± 1.8
17	7.5	72	−80	127.8% ± 0.6
18	7.5	72	4	52.9% ± 1.8
19	10	72	−80	108% ± 1.5
20	10	72	4	54.4% ± 4.8
21	12.5	72	−80	117.3% ± 3.9
22	12.5	72	4	42.1% ± 0.9

**Table 3:** Statistical coefficients of hMBCOMT storage stability optimization.

Output	R^2^	Adjust R^2^	Predicted R^2^	Adequate Precision
% Activity recovery	0.9770	0.9629	0.9074	27.056

**Table 4:** ANOVA analysis for a response surface quadratic model for the percentage of hMBCOMT activity recovery. *p*-value < 0.05 is considered significant.

Source	Sum of Squares	df	Mean Square	F Value	*p*-Value
Model	18,053.09	8	2256.64	69.15	0.0001
Concentration of [Ch][DHP] (A)	733.83	1	733.83	22.49	0.0004
Time (B)	3641.83	1	3641.83	111.59	<0.0001
Temperature (C)	12,871.50	1	12,871.50	394.41	<0.0001
AB	38.59	1	38.59	1.18	0.2966
AC	28.09	1	28.09	0.86	0.3704
BC	660.83	1	660.83	20.25	0.006
A^2^	0.073	1	0.072	2.245 × 10^−3^	0.9629
B^2^	74.02	1	74.02	2.27	0.1560
Residual	424.25	13	32.63	-	-
Lack of Fit	377.37	4	41.93	3.58	0.1160

**Table 5:** Predicted outputs for optimal point. CI—Confidence Interval.

Output	Predicted Mean	SE Mean	95% CI Low	95% CI High	SE Predicted	95% PI Low	95% PI High
% Activity recovery	144.884	3.71267	136.787	152.961	6.83244	130.123	159.645

**Table 6:** hMBCOMT stabilizers features and concentrations.

**Stabilizer**	**Feature**	**Concentration Range**
Cysteine	Stabilization of disulfide bonds	5 to 40 mM
Trehalose	Thermal stabilizers/promote protein folding and refolding	5 to 40 mM
Glycerol	Cryo-protector	5 to 40%
[Ch][DHP]	Thermal stabilizer/aggregation behavior	5–125 mM
[Ch]Cl		5–500 mM
[C12mim]Cl		5–500 mM
[C4mim]Cl		5–500 mM

## References

[1] Antonini A., Abbruzzese G., Barone P., Bonuccelli U., Lopiano L., Onofrj M., Zappia M., Quattrone A. (2008). COMT inhibition with tolcapone in the treatment algorithm of patients with Parkinson’s disease (PD): Relevance for motor and non-motor features. Neuropsychiatr. Dis. Treat..

[2] Boivin S., Kozak S., Meijers R. (2013). Optimization of protein purification and characterization using Thermofluor screens. Protein Expr. Purif..

[3] Apud J.A., Weinberger D.R. (2007). Treatment of cognitive deficits associated with schizophrenia: Potential role of catechol-O-methyltransferase inhibitors. CNS Drugs.

[4] Müller T. (2015). Catechol-O-methyltransferase inhibitors in Parkinson’s disease. Drugs.

[5] Pfeiffer E., Wefers D., Hildebrand A.A., Fleck S.C., Metzler M. (2013). Catechol metabolites of the mycotoxin zearalenone are poor substrates but potent inhibitors of catechol-O-methyltransferase. Mycotoxin Res..

[6] Yager J.D. (2012). Catechol-O-methyltransferase: Characteristics, polymorphisms and role in breast cancer. Drug Discov. Today. Dis. Mech..

[7] Ji Y., Olson J., Zhang J., Hildebrandt M., Wang L., Ingle J., Fredericksen Z., Sellers T., Miller W., Dixon J.M. (2008). Breast cancer risk reduction and membrane-bound catechol O-methyltransferase genetic polymorphisms. Cancer Res..

[8] Zhu B.T. (2002). On the mechanism of homocysteine pathophysiology and pathogenesis: A unifying hypothesis. Histol. Histopathol..

[9] Bai H.W., Shim J.Y., Yu J., Zhu B.T. (2007). Biochemical and molecular modeling studies of the O-methylation of various endogenous and exogenous catechol substrates catalyzed by recombinant human soluble and membrane-bound catechol-O-methyltransferases. Chem. Res. Toxicol..

[10] Cotton N.J., Stoddard B., Parson W.W. (2004). Oxidative inhibition of human soluble catechol-O-methyltransferase. J. Biol. Chem..

[11] Santos F.M., Pedro A.Q., Soares R.F., Martins R., Bonifácio M.J., Queiroz J.A., Passarinha L.A. (2013). Performance of hydrophobic interaction ligands for human membrane-bound catechol-O-methyltransferase purification. J. Sep. Sci..

[12] Vagenende V., Yap M.G., Trout B.L. (2009). Mechanisms of protein stabilization and prevention of protein aggregation by glycerol. Biochemistry.

[13] Pedro A.Q., Pereira P., Bonifácio M.J., Queiroz J.A., Passarinha L.A. (2015). Purification of Membrane-Bound Catechol-O-Methyltransferase by Arginine-Affinity Chromatography. Chromatographia.

[14] Pedro A.Q., Gonçalves A.M., Queiroz J.A., Passarinha L.A. (2018). Purification of Histidine-Tagged Membrane-Bound Catechol-O-Methyltransferase from Detergent-Solubilized Pichia pastoris Membranes. Chromatographia.

[15] Correia F.F., Santos F.M., Pedro A.Q., Bonifácio M.J., Queiroz J.A., Passarinha L.A. (2014). Recovery of biological active catechol-O-methyltransferase isoforms from Q-sepharose. J. Sep. Sci..

[16] Galamba N. (2012). Mapping structural perturbations of water in ionic solutions. J. Phys. Chem. B.

[17] Schröder C. (2017). Proteins in Ionic Liquids: Current Status of Experiments and Simulations. Top. Curr. Chem..

[18] Zhang Y., Cremer P.S. (2010). Chemistry of Hofmeister anions and osmolytes. Annu. Rev. Phys. Chem..

[19] Leibly D.J. (2012). Stabilizing Additives Added during Cell Lysis Aid in the Solubilization of Recombinant Proteins. PLoS ONE.

[20] Kiritsi M.N., Fragoulis E.G., Sideris D.C. (2012). Essential cysteine residues for human RNase kappa catalytic activity. FEBS J..

[21] Liu T., Wang Y., Luo X., Li J., Reed S.A., Xiao H., Young T.S., Schultz P.G. (2016). Enhancing protein stability with extended disulfide bonds. Proc. Natl. Acad. Sci. USA.

[22] Castro G.R. (2000). Properties of soluble alpha-chymotrypsin in neat glycerol and water. Enzym. Microb. Technol..

[23] Naushad M., Alothman Z.A., Khan A.B., Ali M. (2012). Effect of ionic liquid on activity, stability, and structure of enzymes: A review. Int. J. Biol. Macromol..

[24] Rogers R.D., Seddon K.R. (2003). Ionic Liquids—Solvents of the Future?. Science.

[25] Han Q., Brown S.J., Drummond C.J., Greaves T.L. (2022). Protein aggregation and crystallization with ionic liquids: Insights into the influence of solvent properties. J. Colloid Interface Sci..

[26] Ventura S.P., e Silva F.A., Gonçalves A.M., Pereira J.L., Gonçalves F., Coutinho J.A. (2014). Ecotoxicity analysis of cholinium-based ionic liquids to Vibrio fischeri marine bacteria. Ecotoxicol. Environ. Saf..

[27] Fujita K., Forsyth M., MacFarlane D.R., Reid R.W., Elliott G.D. (2006). Unexpected improvement in stability and utility of cytochrome c by solution in biocompatible ionic liquids. Biotechnol. Bioeng..

[28] Fujita K., MacFarlane D.R., Forsyth M., Yoshizawa-Fujita M., Murata K., Nakamura N., Ohno H. (2007). Solubility and stability of cytochrome c in hydrated ionic liquids: Effect of oxo acid residues and kosmotropicity. Biomacromolecules.

[29] Vrikkis R.M., Fraser K.J., Fujita K., Macfarlane D.R., Elliott G.D. (2009). Biocompatible ionic liquids: A new approach for stabilizing proteins in liquid formulation. J. Biomech. Eng..

[30] Chen X., Ji Y., Wang J. (2010). Improvement on the crystallization of lysozyme in the presence of hydrophilic ionic liquid. Analyst.

[31] Pusey M.L., Paley M.S., Turner M.B., Rogers R.D. (2007). Protein Crystallization Using Room Temperature Ionic Liquids. Cryst. Growth Des..

[32] Otrelo-Cardoso A.R., Schwuchow V., Rodrigues D., Cabrita E.J., Leimkühler S., Romão M.J., Santos-Silva T. (2014). Biochemical, stabilization and crystallization studies on a molecular chaperone (PaoD) involved in the maturation of molybdoenzymes. PLoS ONE.

[33] Swatloski R.P., Spear S.K., Holbrey J.D., Rogers R.D. (2002). Dissolution of Cellose with Ionic Liquids. J. Am. Chem. Soc..

[34] Lange C., Patil G., Rudolph R. (2005). Ionic liquids as refolding additives: N′-alkyl and N′-(omega-hydroxyalkyl) N-methylimidazolium chlorides. Protein Sci..

[35] Buchfink R., Tischer A., Patil G., Rudolph R., Lange C. (2010). Ionic liquids as refolding additives: Variation of the anion. J. Biotechnol..

[36] Golovanov A.P., Hautbergue G.M., Wilson S.A., Lian L.Y. (2004). A simple method for improving protein solubility and long-term stability. J. Am. Chem. Soc..

[37] Barreca D., Lagana G., Magazu S., Migliardo F., Gattuso G., Bellocco E. (2014). FTIR, ESI-MS, VT-NMR and SANS study of trehalose thermal stabilization of lysozyme. Int. J. Biol. Macromol..

[38] Tilgmann C., Ulmanen I. (1996). Purification methods of mammalian catechol-O-methyltransferases. J. Chromatogr. B Biomed. Appl..

[39] Pedro A.Q., Oppolzer D., Bonifácio M.J., Maia C.J., Queiroz J.A., Passarinha L.A. (2015). Evaluation of Mut(S) and Mut⁺ Pichia pastoris strains for membrane-bound catechol-O-methyltransferase biosynthesis. Appl. Biochem. Biotechnol..

[40] Costa S.R., Bonifacio M.J., Queiroz J.A., Passarinha L.A. (2011). Analysis of hSCOMT adsorption in bioaffinity chromatography with immobilized amino acids: The influence of pH and ionic strength. J. Chromatogr. B Anal. Technol. Biomed. Life Sci..

[41] Pedro A., Soares R., Oppolzer D., Santos F., Rocha L., Gonçalves A., Bonifacio M., Queiroz J., Gallardo E., Passarinha L. (2014). An improved HPLC method for quantification of metanephrine with coulometric detection. J. Chromatogr. Sep. Tech..

[42] Egorova K.S., Gordeev E.G., Ananikov V.P. (2017). Biological Activity of Ionic Liquids and Their Application in Pharmaceutics and Medicine. Chem. Rev..

[43] Fujita K. (2019). Ionic Liquids as Stabilization and Refolding Additives and Solvents for Proteins. Adv. Biochem. Engin./Biotechnol..

[44] Zhao H. (2016). Protein Stabilization and Enzyme Activation in Ionic Liquids: Specific Ion Effects. J. Chem. Technol. Biotechnol..

[45] Kumar A., Bisht M., Venkatesu P. (2017). Biocompatibility of ionic liquids towards protein stability: A comprehensive overview on the current understanding and their implications. Int. J. Biol. Macromol..

[46] Fujita K., Kajiyama M., Liu Y., Nakamura N., Ohno H. (2016). Hydrated ionic liquids as a liquid chaperon for refolding of aggregated recombinant protein expressed in Escherichia coli. Chem. Comm..

[47] Reslan M., Ranganathan V., Macfarlane D.R., Kayser V. (2018). Choline ionic liquid enhances the stability of Herceptin^®^ (trastuzumab). Chem. Comm..

[48] Tarver C.L., Yuan Q., Pusey M.L. (2021). Ionic Liquids as Protein Crystallization Additives. Crystals.

[49] Yamamoto E., Yamaguchi S., Nagamune T. (2011). Protein Refolding by N-Alkylpyridinium and N-Alkyl-N-methylpyrrolidinium Ionic Liquids. Appl. Biochem. Biotechnol..

[50] Zhao H. (2010). Methods for stabilizing and activating enzymes in ionic liquids—A review. J. Chem. Technol. Biotechnol..

[51] Park S., Kazlauskas R.J. (2003). Biocatalysis in ionic liquids—Advantages beyond green technology. Curr. Opin. Biotechnol..

[52] Fukaya Y., Iizuka Y., Sekikawa K., Ohno H. (2007). Bio ionic liquids: Room temperature ionic liquids composed wholly of biomaterials. Green Chem..

[53] Jerome L.M., Well A.D., Lorch R.F. (2010). Research Design and Statistical Analysis.

[54] Montgomery D.C. (2019). Design and Analysis of Experiments.

[55] Carlson R. (2001). Design of Experiments, Principles and Applications, L. Eriksson, E. Johansson, N. Kettaneh- Wold, C. Wikström and S. Wold, Umetrics AB, Umeå Learnways AB, Stockholm, 2000, ISBN 91-973730-0-1, xii + 329 pp. J. Chemom..

